# Rhythmicity in Mice Selected for Extremes in Stress Reactivity: Behavioural, Endocrine and Sleep Changes Resembling Endophenotypes of Major Depression

**DOI:** 10.1371/journal.pone.0004325

**Published:** 2009-01-29

**Authors:** Chadi Touma, Thomas Fenzl, Jörg Ruschel, Rupert Palme, Florian Holsboer, Mayumi Kimura, Rainer Landgraf

**Affiliations:** 1 Max Planck Institute of Psychiatry, Munich, Germany; 2 Department of Biomedical Sciences, University of Veterinary Medicine, Vienna, Austria; James Cook University, Australia

## Abstract

**Background:**

Dysregulation of the hypothalamic-pituitary-adrenal (HPA) axis, including hyper- or hypo-activity of the stress hormone system, plays a critical role in the pathophysiology of mood disorders such as major depression (MD). Further biological hallmarks of MD are disturbances in circadian rhythms and sleep architecture. Applying a translational approach, an animal model has recently been developed, focusing on the deviation in sensitivity to stressful encounters. This so-called ‘stress reactivity’ (SR) mouse model consists of three separate breeding lines selected for either high (HR), intermediate (IR), or low (LR) corticosterone increase in response to stressors.

**Methodology/Principle Findings:**

In order to contribute to the validation of the SR mouse model, our study combined the analysis of behavioural and HPA axis rhythmicity with sleep-EEG recordings in the HR/IR/LR mouse lines. We found that hyper-responsiveness to stressors was associated with psychomotor alterations (increased locomotor activity and exploration towards the end of the resting period), resembling symptoms like restlessness, sleep continuity disturbances and early awakenings that are commonly observed in melancholic depression. Additionally, HR mice also showed neuroendocrine abnormalities similar to symptoms of MD patients such as reduced amplitude of the circadian glucocorticoid rhythm and elevated trough levels. The sleep-EEG analyses, furthermore, revealed changes in rapid eye movement (REM) and non-REM sleep as well as slow wave activity, indicative of reduced sleep efficacy and REM sleep disinhibition in HR mice.

**Conclusion/Significance:**

Thus, we could show that by selectively breeding mice for extremes in stress reactivity, clinically relevant endophenotypes of MD can be modelled. Given the importance of rhythmicity and sleep disturbances as biomarkers of MD, both animal and clinical studies on the interaction of behavioural, neuroendocrine and sleep parameters may reveal molecular pathways that ultimately lead to the discovery of new targets for antidepressant drugs tailored to match specific pathologies within MD.

## Introduction

The rotation of the earth exposes all organisms to a daily change in light intensity and virtually all species have adapted their lifestyles to cycles of 24 hours [1]. These daily rhythms are endogenously generated and are synchronised to external time cues in order to ensure that bodily processes are carried out at the appropriate, optimal time of day or night [2], [3]. In mammals, the suprachiasmatic nuclei in the anterior-ventral hypothalamus are the principal oscillator coordinating many physiological and behavioural functions, including the circadian rhythms of body temperature, hormone secretion (e.g. melatonin, luteinising hormone, growth hormone) and sleep-wake behaviour [2]–[4]. The activity of the hypothalamic-pituitary-adrenal (HPA) axis is also characterised by a prominent circadian rhythm with peak glucocorticoid (GC) secretion occurring shortly before the onset of an animal's activity period and trough levels during the beginning of the resting period [4], [5]. This daily variation of GC concentration is critical for homeostatic regulation of metabolic, cardiovascular and neural processes, and a bidirectional interaction between sleep and the HPA system has been well established [6]–[8].

Interestingly, the sleep-endocrine regulation is critically influenced by brain areas, which also play an important role in the pathophysiology of affective disorders such as major depression (MD) [6]–[10]. These include the hypothalamus, particularly the paraventricular nucleus, but also limbic areas such as the hippocampus and the amygdala, the prefrontal cortex as well as afferent brain nuclei, in particular the locus coeruleus and the raphe nuclei [6]–[11]. Therefore, it is not surprising that sleep disturbances are among the most common symptoms of MD [6], [7], [9]–[12]. Compared to healthy subjects, electroencephalogram (EEG) recordings, which allow to objectively assess sleep alterations, revealed that MD patients often suffer from insomnia and sleep fragmentation (i.e. increased time to fall asleep, frequent awakenings and early morning awakenings, some hours earlier than desired, with difficulty returning to sleep). They also show a reduced latency to the first episode of rapid eye movement (REM) sleep, an increased proportion of REM sleep (increased REM density and intensity) and reduced slow-wave activity (SWA) during non-REM (NREM) sleep [6], [7], [11], [12].

Another biological hallmark of MD is the dysregulation of the HPA axis (hyper- or hypo-activity), largely involving pathological alterations in the corticotrophin-releasing hormone (CRH) system (for reviews see [6], [9], [10], [13]–[20]). Therefore, sleep-EEG and stress hormone alterations were also among the first biological changes reported in MD [21]–[23]. Common neuroendocrine symptoms of severely depressed patients include a flattened diurnal rhythm of GC secretion (in particular, elevated trough levels have been observed [24]–[27], elevated plasma and 24-h urinary GC concentrations (hypercortisolism) and adrenal hyperplasia. Furthermore, dysfunctional GC receptor-mediated negative feedback regulation of the HPA axis and changes in vasopressin and CRH responsiveness have frequently been described [9], [13], [15]–[20]. However, it is increasingly acknowledged that the diagnosis of MD encompasses patients who do not necessarily share the same disease biology, supporting the concept of different subtypes of depression [7], [10], [15], [17], [28]. For instance, HPA axis overdrive, related to an enhanced secretion of CRH and an impaired negative feedback via GC receptors, is most consistently observed in patients with melancholic depression. These patients also show the most pronounced sleep-EEG alterations, including disrupted sleep, decreased SWA, short REM sleep latency and high REM sleep density. In contrast, patients presenting with the so-called atypical subtype of depression are characterised by markedly reduced activity of the HPA axis, while sleep-EEG data suggest that SWA is not reduced and REM sleep parameters are not considerably altered in these patients [7], [15], [17], [28], [29].

Based on the vital link between stress sensitivity and the development of MD [9], [13], [16], [18], [20], a new, genetic animal model has been recently established at the Max Planck Institute of Psychiatry, focusing on alterations in HPA axis reactivity [30]. This so-called ‘stress reactivity’ (SR) mouse model consists of three separate breeding lines selected for either high (HR), intermediate (IR), or low (LR) corticosterone increase in response to a moderate psychological stressor (15-min restraint). Significant differences in the reactivity of the HPA axis between HR, IR and LR mice were already found in the first generation of the selective breeding process and proved to be a highly heritable trait, i.e. the respective phenotype was confirmed across all subsequent generations and could even be increased by assortative breeding [30]. Moreover, results of an extensive behavioural test battery applied to the selected mouse lines as well as neuroendocrine characterisation experiments revealed several phenotypic similarities with changes observed in depressive patients [30]. In general, HR animals were relatively hyperactive in some behavioural paradigms, resembling symptoms of restlessness and agitation often seen in melancholic depression. LR mice, on the other hand, showed more passive-aggressive coping styles, corresponding to signs of retardation and retreat observed in atypical depression.

As outlined above, HPA axis functioning plays a critical role for the regulation of sleep and activity rhythms. Therefore, the aim of this study was to combine the analysis of behavioural and HPA axis rhythmicity with sleep-EEG recordings in the HR/IR/LR mouse lines, in order to provide a more comprehensive picture of endophenotypes associated with increased or decreased stress reactivity. Thus, we intended to further contribute to the validation of the SR mouse model as a promising tool to elucidate molecular genetic, neuroendocrine and behavioural parameters associated with altered HPA axis reactivity.

## Methods

### Animals and general housing conditions

All animals used in this study derived from the seventh generation (Gen VII) of the ‘stress reactivity’ (SR) mouse model. As outlined above, this model consists of three independent mouse lines selectively bred for either high (HR), intermediate (IR) or low (LR) reactivity of the HPA axis (for a detailed description of the model see [30]).

Details about housing conditions, age, and the number of mice used in each experiment are given in the respective sections (see below). In general, from weaning at the age of about four weeks all animals were housed in same-sex groups of two to four mice in transparent polycarbonate cages (standard Macrolon cages type III, 38×22×15 cm^3^) with wood chips as bedding and wood shavings as nesting material (Product codes: LTE E-001 and NBF E-011, ABEDD - LAB and VET Service GmbH, Vienna, Austria). The animal housing room as well as the experimental rooms were maintained under standard laboratory conditions (light-dark cycle: 12 : 12 h, lights on at 8 a.m.; temperature: 22±1°C; relative humidity: 55±10%). Commercial mouse diet (Altromin No. 1324, Altromin GmbH, Lage, Germany) and bottled tap water were available *ad libitum*.

The presented work complies with current regulations covering animal experimentation in Germany and the EU (European Communities Council Directive 86/609/EEC). All experiments were announced to the appropriate local authority and were approved by the ‘Animal Welfare Officer’ of the Max Planck Institute of Psychiatry (Az. 55.2-1-54-2531-64-07 and Az. 209.1/211-33/04).

### Stress reactivity testing and selection of experimental animals

Routinely, all animals of each breeding generation of the SR mouse model are subjected to a so-called ‘stress reactivity test’ (SRT) performed at around eight weeks of age. Details about the test procedure and subsequent analyses are described by Touma and colleagues [30]. Briefly, the SRT consists of a 15-min restraint period and two tail blood samplings immediately before and after exposure to the stressor. All animals are tested in the first hours of the light phase (between 9 a.m. and 11 a.m.), i.e. during the trough of the circadian rhythm of GC secretion. From the collected ‘initial’ and ‘reaction’ blood samples, corticosterone concentrations are determined by radioimmunoassay, quantifying the reactivity of the HPA axis as corticosterone increase in response to a moderate psychological stressor.

According to the outcome of this SRT, 36 male mice (12 of each breeding line) of Gen VII were selected as experimental animals, showing a high, intermediate or low corticosterone increase, characteristic of the neuroendocrine stress response phenotype of the HR, IR and LR breeding line, respectively [30].

### Behavioural activity rhythms

To monitor the undisturbed behavioural activity rhythm of the animals, we used a self-made device (‘System for Automatic Measurement of Laboratory Animals’ Behaviour' *SAMLAB*) to automatically track resting *versus* motor activity, explorative behaviours as well as feeding and drinking activities in the home cage. This was achieved by placing two computer-connected metal frames equipped with 48 infrared light barriers (32×16 photo electric sensors) positioned at a distance of about 1 cm around each cage (standard Macrolon cage type III with a special bedding material: Rehofix maize granulate, MK 2000, round particle size: 1.7–2.2 mm, absorption capacity: 2.0 l/kg), with the first frame 3 cm above ground level and the other 5 cm higher. Thereby, the cage floor as well as the upper level of the cage were divided into a grid of 1.25×1.5 cm^2^, enabling the detection of the position of the mouse and the calculation of its movements according to light beam breaks using a customized software (construction and programming of *SAMLAB* by Oleg Dolgov). Motor activity was defined as non-resting, i.e. when the mouse was moving and activating changing sets of light barriers. Explorative behaviours such as rearing and climbing on the cage lid could also be detected, i.e. when the mouse activated light barriers at the ground and upper level simultaneously (rearing) or only showed movement in the upper grid of light barriers (climbing). In order to avoid potentially confounding influences of human activities in the housing room on the animals activity rhythm, the whole activity monitoring setup was built into a soundproof cabinet equipped with an autonomic ventilation, temperature, humidity and light control system (set to the same conditions as in the housing room; see above). Glass doors allowed inspections of the test animals without disturbing the measurements.

Each mouse (12 HR, IR and LR males, respectively, about 10 weeks of age; see above) was single housed for at least two weeks before being put into the activity monitoring device and was kept in the light barrier monitored cages for one week with minimal disturbance from outside. The first three days in the apparatus were regarded as a habituation period and, therefore, only the data of the last four days were analysed. The ‘time spent active’ (motor activity and explorative behaviours; see definitions above) was continuously recorded during the entire 24-hour light-dark cycle and was averaged for each individual in hourly intervals over the four recording days, resulting in a mean activity pattern for each mouse.

### Diurnal rhythm of glucocorticoid secretion

In order to accurately follow the natural diurnal rhythm of glucocorticoid secretion in HR, IR and LR mice without interfering with the activation of the HPA axis by repeated handling and blood sampling, a non-invasive technique to monitor adrenocortical activity by measuring corticosterone metabolites (CM) in the faeces of mice was applied [31], [32]. This technique of glucocorticoid metabolite quantification in faecal samples has been established in a large number of species (for review see [33]) and has been extensively validated for laboratory mice [31], [32].

The same 36 HR/IR/LR males (12 of each breeding line) that were characterised with respect to their behavioural activity rhythms (see above) were also used in this experiment (after two weeks of normal housing in standard cages). For a period of 48 hours, faecal samples were collected quantitatively in short sampling intervals of two hours and stored at −20°C until analysis of CM (see below). To facilitate individual sampling and quantitative collection of all voided faeces without handling the animal, the method described by Touma and colleagues was used [31], [32]. Briefly, the mice were housed individually in stainless steel wire cages (38×22×15 cm^3^), which were placed in standard Macrolon cages of the same size. All excreta dropped through the bars of the wire cage and could easily be collected from the floor of the lower cage, which was completely covered with filter paper that immediately absorbed the urine. To habituate the mice to this sampling procedure and to being housed in wire cages, the animals were already placed into this housing system three days prior to the beginning of the experiment and samples were collected in 12 hour intervals during this time. Since mice are nocturnal animals and their steroid excretion pattern is known to be influenced by their activity [32], all sample collections performed during the dark phase of the light-dark cycle were conducted under dimmed lighting conditions (less than 5 lux) to avoid disturbing the animals' natural activity pattern.

The collected faecal samples were analyzed for immunoreactive CM using a 5α-pregnane-3β,11β,21-triol-20-one enzyme-immunoassay (EIA). Details regarding development, biochemical characteristics, and biological validation of this assay are described by Touma and colleagues [31], [32]. Before EIA analysis, the faecal samples were homogenized and aliquots of 0.05 g were extracted with 1 ml of 80% methanol. A detailed description of the assay performance has been published elsewhere [32]. Briefly, the EIA used a double-antibody technique and was performed on anti-rabbit-IgG-coated microtitre plates. After overnight incubation (at 4°C) of standards (range: 0.8–200 pg/well) and samples with steroid antibody and biotinylated label, the plates were emptied, washed and blotted dry, before a streptavidin horseradish peroxidase conjugate was added. After 45 minutes incubation time, plates were emptied, washed, and blotted dry. The substrate (tetramethylbenzidine) was added and incubated for another 45 minutes at 4°C before the enzymatic reaction was stopped with 1 mol/l sulphuric acid. Then, the optical density (at 450 nm) was recorded with an automatic plate reader and the hormone concentrations were calculated. The intra- and inter-assay coefficients of variation were 8.8% and 13.4%, respectively.

For each individual, CM concentrations of the two corresponding sampling intervals during the 48 hour sampling period were averaged, yielding a mean diurnal pattern of glucocorticoid secretion for each mouse.

### Sleep recordings

To study the sleep patterns and quality of HR, IR and LR mice, EEG recordings were performed with a subset of animals (N = 8 males per line) from the experiments described above. All animals were housed individually in customized recording cages (26×26×35 cm^3^) located in sound-attenuated chambers kept at constant laboratory conditions (22°C ±1°C, 12 : 12 h light-dark cycles, lights on at 10 a.m.). Food and water were available *ad libitum*.

Surgical procedures were performed under isoflurane/oxygen anaesthesia using a custom-made vaporizing device. At the beginning of the surgery, each animal also received atropinesulfate (0.05 mg/kg BW) and meloxicam (0.5 mg/kg BW) subcutaneously for cardiovascular stabilisation and analgesia, respectively. The animals were positioned in a stereotactic frame and four epidural EEG and two intramuscular electromyogram (EMG) electrodes were implanted. Briefly, the skin and muscles overlaying the skull were cut rostro-caudally along the midline, drawn to the sides and kept in place using small retractors. To insert the EEG electrodes, four small holes (diameter: 200 µm) were drilled into the skull. Two electrodes were placed bilaterally at the frontal region of the cortex, one reference-electrode was placed at the right parietal area, and the ground electrode was inserted at the left parietal area. All four electrodes were fixed with dental cement to the skull. Additionally, two bilateral EMG electrodes were embedded laterally of the spine into the neck muscles. All electrodes were composed of gold wire with ball-shaped endings, soldered to a small standard printed circuit board connector. In order to provide more stability to the assembly on the skull for chronic recording, two additional small holes were drilled for mounting jeweller's screws that were also framed with dental cement and glued together with the electrodes and the connector.

After surgery, the animals were allowed to recover for two weeks in the recording cages before two successive 23-hour recording sessions of EEG and EMG signals were performed. During recovery and recording each mouse was attached to a recording cable, which was connected to a swivel system allowing relatively free movement of the animals.

EEG and EMG signals were fed online into a preamplifier (1000×, custom made) and a main amplifier (10×, custom made). The EEG signals were analogue band-pass filtered (0.5–29 Hz, filter frequency roll off 48 dB/octave) and digitized at a sampling rate of 64 Hz (AD board, NI PCI-6070, National Instruments, Austin, USA). Root mean square was applied to all non-filtered EMG signals before its digital conversion (64 Hz). The vigilance states ‘wake’, ‘non-rapid eye movement sleep’ (NREM sleep) and ‘rapid eye movement sleep’ (REM sleep) were scored on a LabVIEW-based scoring program (SEA, Köln, Germany) semi-automatically with a Fast Fourier Transformation algorithm spectral analysis and could be corrected manually, if necessary (the scoring technique was validated beforehand). The frequency bands were as follows: δ (0.5–5 Hz), θ (6–9 Hz), α (10–15 Hz), η (16–22.5 Hz) and β (23–31.75 Hz). A detailed description of the scoring procedure is described elsewhere [34]. Slow wave activity (SWA, NREM sleep frequency bands: 0.5–15 Hz; SWA frequency bands: 0.5–4 Hz in 0.5 Hz steps) was calculated from the total amount of NREM sleep across the 23-hour recording time in one hour means.

### Statistical Analysis

Since a normal distribution and variance homogeneity of the data could not always be assumed, analyses were exclusively performed using non-parametric statistics [35]. All tests were applied two-tailed and were calculated using the software package SPSS (version 16.0). ANOVA on ranks (Friedman-test) was used to evaluate differences between more than two dependent (related) samples. Two independent samples were compared using the Mann-Whitney U-test (MWU-test), while differences between more than two independent samples were calculated with the Kruskal-Wallis H-test (KWH-test). In the case of significant variation proved by the KWH-test, post-hoc pairwise comparisons between the groups were done using multiple MWU-tests. Spearman's rank-order correlation was calculated to elucidate the degree of association between two variables. As nominal level of significance α = 0.05 was accepted and corrected for post-hoc tests according to the sequential Bonferroni technique [36].

## Results

### HPA axis reactivity

**Figure 1:**
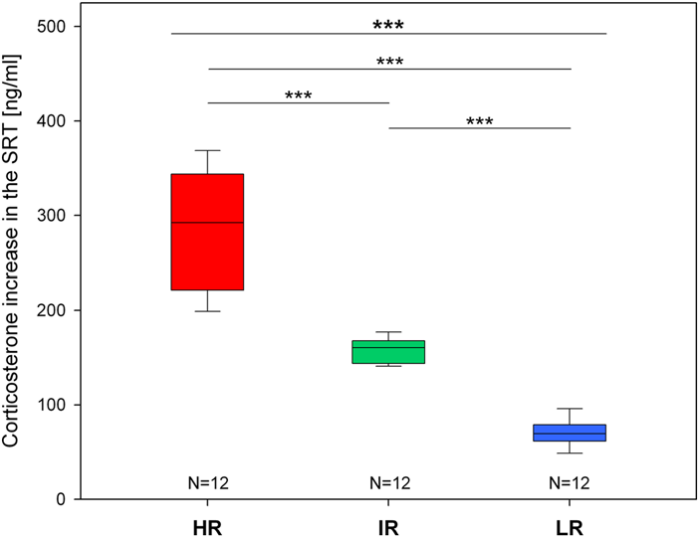
Corticosterone increase in the stress reactivity test (SRT) of the experimental animals selected from the seventh generation of the high (HR), intermediate (IR) and low (LR) reactivity mouse lines. Data are given as box plots showing medians (lines in the boxes), 25% and 75% percentiles (boxes) as well as 10% and 90% percentiles (whiskers). Statistical differences between the three lines (KWH-test, for details see text) are given at the top of the panel and results of the pairwise group comparisons (post-hoc MWU-tests) are indicated below (Bonferroni corrected p<0.001 ***).

As expected, the experimental animals selected from generation VII of the HR, IR and LR breeding lines differed significantly regarding their corticosterone increase in the SRT (KWH-test: N = 12 for each line, H = 31.1, df = 2, p<0.001; see Fig. 1). HR mice showed a very much exaggerated stress response, while compared to IR animals the secretion of corticosterone was strongly reduced in LR mice (see Fig. 1).

### Behavioural activity rhythms

**Figure 2:**
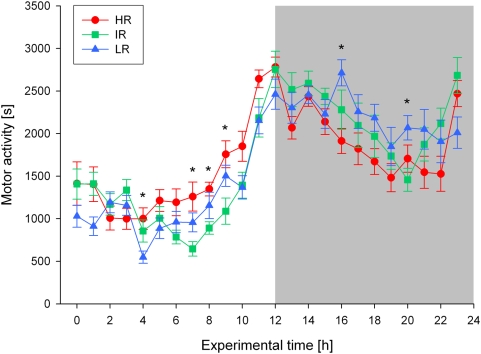
Distribution of motor activity over the 24-h light-dark cycle in high (HR), intermediate (IR), and low (LR) reactivity males from generation VII. Data are given as means±SEM for each line. Statistical differences between the three lines are indicated by asterisks (KWH-tests, for details see text, p<0.05 *). The dark phase of the light-dark cycle is indicated by the shaded area.

The behavioural activity rhythms also differed significantly between the three breeding lines. Although a clear pattern of increased motor activity during the dark phase and less activity during the light phase could be observed in all mouse lines (Friedman-tests: N = 12 for each line, Chi_r_^2^ = 134.1–142.7, df = 23, all p<0.001; see Fig. 2), at several time points during the 24-hour light-dark cycle significant differences in motor activity were found between HR, IR and LR animals (KWH-tests: N = 12 for each line, experimental time points: 4, 7, 8, 9, 16, 20, H = 6.4–8.9, df = 2, all p<0.05; see Fig. 2). In particular, in the second half of the light phase, i.e. some hours before the light-dark transition, HR mice were clearly more active than IR and LR mice, while the latter did not differ significantly from each other in their overall activity pattern (see Fig. 2).

**Figure 3:**
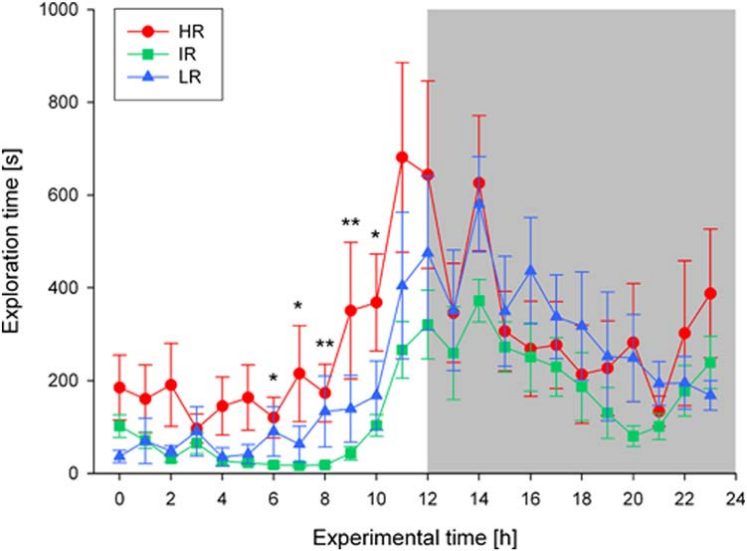
Distribution of explorative behaviour over the 24-h light-dark cycle in high (HR), intermediate (IR), and low (LR) reactivity males from generation VII. Data are given as means±SEM for each line. Statistical differences between the three lines are indicated by asterisks (KWH-tests, for details see text, p<0.05 *, p<0.01 **). The dark phase of the light-dark cycle is indicated by the shaded area.

A similar picture emerged for the distribution of explorative behaviours such as rearing and climbing. Again, all three mouse lines showed a significant variation over the day regarding the time spent exploring the cage (Friedman-tests: N = 12 for each line, Chi_r_^2^ = 135.5–149.5, df = 23, all p<0.001; see Fig. 3). However, the increase of explorative activities shortly before the light-dark transition was much more pronounced and advanced by some hours in HR mice compared to the other two lines, resulting in significant differences at several time points during the light phase (KWH-tests: N = 12 for each line, experimental time points: 6, 7, 8, 9, 10, H = 6.1–12.6, df = 2, all p<0.05; see Fig. 3).

### Diurnal rhythm of glucocorticoid secretion

**Figure 4:**
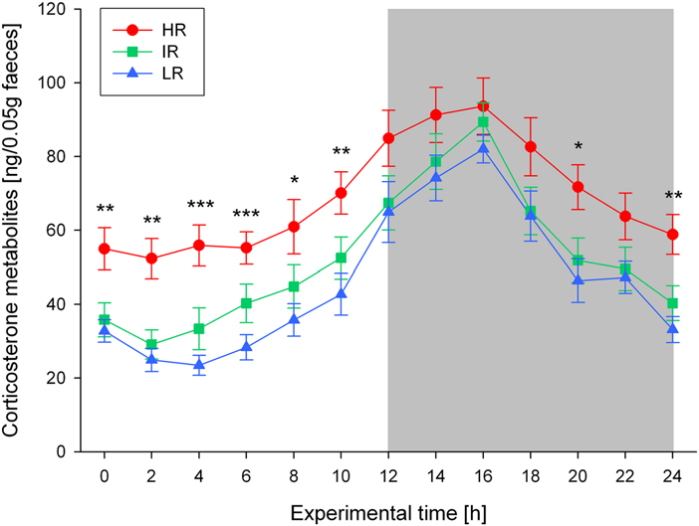
Diurnal variation of immunoreactive corticosterone metabolites (CM) in faecal samples of high (HR), intermediate (IR), and low (LR) reactivity males from generation VII over the 24-h light-dark cycle. Data are given as means±SEM for each line. Statistical differences between the three lines are indicated by asterisks (KWH-tests, for details see text, p<0.05 *, p<0.01 **, p<0.001 ***). The dark phase of the light-dark cycle is indicated by the shaded area.

Regarding the diurnal rhythm of glucocorticoid secretion, all three mouse lines showed a significant variation of CM concentrations over the 24-hour light-dark cycle (Friedman-tests: N = 12 for each line, Chi_r_^2^ = 100.9–110.5, df = 12, all p<0.001; see Fig. 4). Overall, highest concentrations were measured during the dark phase (peaking around midnight), while relatively low CM levels were observed during the light phase. Comparing the concentrations of excreted CM between HR, IR and LR animals across the day, however, revealed significant differences at several sampling time points during the light as well as during the dark phase (KWH-tests: N = 12 for each line, experimental time points: 0, 2, 4, 6, 8, 10, 20, 24, H = 8.4–18.2, df = 2, all p<0.05; see Fig. 4). In general, HR mice showed distinctly and significantly higher CM concentrations than IR and LR animals, in particular during the light phase, but not so much at the beginning of the dark phase (see Fig. 4), resulting in a flattened diurnal rhythm of CM excretion (difference between the maximum and minimum CM concentration across the day = Delta means: HR = 53.4, IR = 69.9, LR = 68.2, KWH-test: N = 12 for each line, H = 7.5, df = 2, p<0.05). Furthermore, the area under the curve (AUC) and the mean location (ML) of CM concentrations was significantly higher in HR animals compared to the other two lines, which did not differ significantly from each other (AUC means: HR = 1678.3, IR = 1280.1, LR = 1133.5; ML means: HR = 68.9, IR = 52.2, LR = 46.1; KWH-tests: N = 12 for each line, H = 8.2 and 8.4, df = 2, both p<0.05).

### Sleep recordings

**Figure 5:**
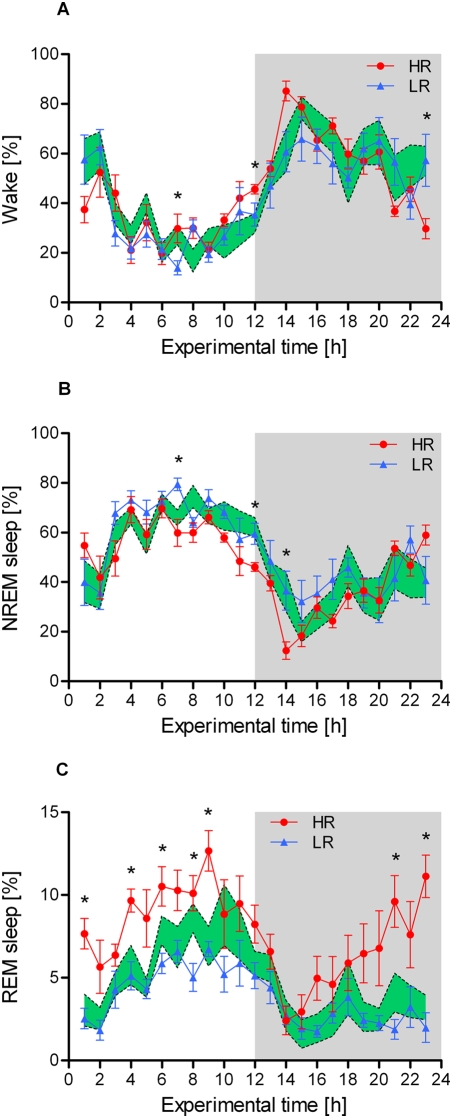
Distribution of vigilance states over the 24-h light-dark cycle in high (HR), intermediate (IR), and low (LR) reactivity males from generation VII. The relative amount of wakefulness, non-rapid eye movement (NREM) sleep and rapid eye movement (REM) sleep are plotted in panel A, B and C, respectively. Data are given as means±SEM for HR and LR mice and as SEM-area for the IR mouse line. Statistical differences between the three lines are indicated by asterisks (KWH-tests, for details see text, p<0.05 *). The dark phase of the light-dark cycle is indicated by the shaded area.

The results of the sleep recording experiment are presented in figure 5. Similar to the behavioural activity patterns described above, the distribution of wakefulness and sleep varied significantly during the course of the day in all three mouse lines, with a larger amount of time spent sleeping in the light phase than in the dark phase. However, at several time points across the light-dark cycle, the relative amount of time the animals spent in either vigilance state (wake, NREM sleep or REM sleep) differed significantly between HR, IR and LR mice.

The amount of wakefulness was higher in HR mice during the second half of the light phase, but significant effects were also found at the end of the dark phase (KWH-tests: N = 8 for each line, experimental time points: 7, 12, 23, H = 6.0–8.4, df = 2, all p<0.05; see Fig. 5A). Similarly, the lines differed significantly in the total amount of NREM sleep (KWH-tests: N = 8 for each line, experimental time points: 7, 12, 14, H = 6.2–9.9, df = 2, all p<0.05; see Fig. 5B). Post-hoc pairwise comparisons revealed that HR mice spent less time in NREM sleep than LR mice (MWU-tests: N = 8 for each line, experimental time points: 7, 12, U = 6–10, all Bonferroni corrected p<0.05; see Fig. 5B). The most pronounced differences between the three lines, however, were found regarding the amount of REM sleep (KWH-tests: N = 8 for each line, experimental time points: 1, 4, 6, 8, 9, 21, 23, H = 8.4–15.8, df = 2, all p<0.05; see Fig. 5C). During the majority of time points in the light phase as well as towards the end of the dark phase, HR mice spent much more time in REM sleep than LR (MWU-tests: N = 8 for each line, experimental time points: 1, 4, 6, 8, 9, 21, 23, U = 0–8, all Bonferroni corrected p<0.05; see Fig. 5C) and IR animals (MWU-tests: N = 8 for each line, experimental time points: 1, 4, 9, 21, 23, U = 1–10, all Bonferroni corrected p<0.05; see Fig. 5C).

**Figure 6:**
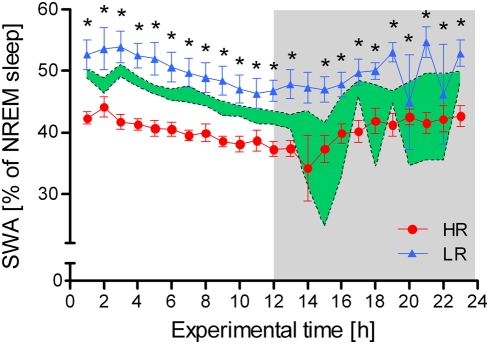
Distribution of the relative amount of slow wave activity (SWA) over the 24-h light-dark cycle in high (HR), intermediate (IR), and low (LR) reactivity males from generation VII. Data are given as means±SEM for HR and LR mice and as SEM-area for the IR mouse line. Statistical differences between the three lines are indicated by asterisks (KWH-tests, for details see text, p<0.05 *). The dark phase of the light-dark cycle is indicated by the shaded area.

Focusing on the SWA within NREM sleep episodes also revealed distinct differences between the three mouse lines at virtually every time point across the light-dark cycle (KWH-tests: N = 8 for each line, experimental time points: 1, 2, 3, 4, 5, 6, 7, 8, 9, 10, 11, 12, 13, 15, 16, 17, 18, 19, 20, 21, 22, 23, H = 4.6–15.0, df = 2, all p<0.05; see Fig. 6). Post-hoc tests confirmed that HR mice showed a clearly and significantly decreased SWA, particularly when compared to LR males (MWU-tests: N = 8 for each line, experimental time points: 1, 3, 4, 5, 6, 7, 8, 9, 10, 11, 12, 13, 15, 16, 17, 19, 20, 21, 23, U = 0–5, all Bonferroni corrected p<0.05, experimental time points: 2, 18, 22, U = 6–8, all Bonferroni corrected p<0.1; see Fig. 6), but also in comparison to IR animals (MWU-tests: N = 8 for each line, experimental time points: 1, 3, 4, 5, 6, 7, 8, 9, 10, 12, 13, 17, 20, 21, 22, 23, U = 0–13, all Bonferroni corrected p<0.05, experimental time points: 15, 16, 18, 19, U = 8–14, all Bonferroni corrected p<0.1; see Fig. 6). At some experimental time points, the amount of SWA was also significantly higher in LR than in IR animals (MWU-tests: N = 8 for each line, experimental time points: 13, 19, 20, 21, U = 2–8, all Bonferroni corrected p<0.05, experimental time point: 12, U = 12, Bonferroni corrected p<0.1; see Fig. 6).

Correlation analysis further revealed in HR mice significant associations between stress reactivity (corticosterone increase in the SRT) and the AUC of excreted CM (r_s_ = 0.783, Bonferroni corrected p<0.01), the proportion of REM sleep (r_s_ = 0.810, Bonferroni corrected p<0.05) and the amount of SWA (r_s_ = −0.738, Bonferroni corrected p<0.05) across the day. That is, animals with a greater corticosterone response in the SRT showed a higher CM excretion profile, a greater increase in REM sleep and a stronger decrease in SWA. For IR and LR animals, however, no such correlations were found.

## Discussion

Clinical studies provide clear evidence for a critical role of circadian rhythm and sleep disturbances in the pathophysiology of mood disorders, which are also closely linked to another biological marker of MD, the dysregulation of the HPA axis (for reviews see [6], [9], [10], [13]–[20]. Applying a selective breeding approach, we developed an animal model that resembles the deviation in sensitivity to stressful encounters [30]. The aim of the present study was to investigate this ‘stress reactivity’ mouse model with respect to the clinically relevant endophenotypes of rhythmicity and sleep disturbances.

We found significant differences between HR, IR and LR mice regarding their circadian rhythm of psychomotor activity and GC secretion as well as pronounced alterations in their sleep-EEG profiles. HR mice for instance showed increased wakefulness, locomotor activity and exploratory behaviours towards the end of the resting period. Moreover, the amplitude of the circadian GC rhythm was reduced due to elevated trough levels and the proportion of REM sleep was clearly increased in these animals. NREM sleep and SWA on the other hand were reduced in comparison to the other two lines. No major rhythmicity differences were found between IR and LR mice, except for a significantly higher proportion of slow wave sleep across the day in LR animals.

In the experiments addressing the behavioural activity rhythms of the animals our results revealed significant differences in the diurnal activity patterns of the three mouse lines. In general, as expected for nocturnal rodents, all animals were more active during the dark phase than during the light phase, but compared to the other two lines, HR mice showed a marked increase in activity towards the end of the light phase, i.e. some hours before the light-dark transition. This increased psychomotor activity during the resting period was found in the analysis of locomotion (see Fig. 2) as well as exploratory behaviours (see Fig. 3) and can be interpreted as resembling the symptoms of sleep fragmentation and early morning awakenings often seen in melancholically depressed patients [6], [7], [12]. This interpretation is also supported by our sleep-EEG data, including a detailed event related analysis (see discussion below). The fact that LR mice did not differ considerably from IR animals with respect to their behavioural activity rhythm is also in accordance with clinical findings, as MD patients with atypical features are not reported to suffer from sleep continuity disturbances or restlessness [7], [37].

Concerning the diurnal variation of HPA axis activity, i.e. the circadian rhythm of GC secretion, similar differences between the three mouse lines were found, as observed for the behavioural rhythms. Again, a clear pattern of increased GC concentrations (measured as faecal CM) during the activity period and relatively low levels during the resting period (including a trough at the beginning of the light phase) were observed in all animals (see Fig. 4). This typical cycle of nadir and peak concentrations is very much in accordance with published data on laboratory rats (plasma samples [38], [39]; faecal samples [40]) and mice (plasma samples [41]–[43]; faecal samples [31], [44], [45]). However, compared to the other two lines, HR mice showed clearly elevated concentrations of faecal CM during the entire light phase as well as at the end of the dark phase, resulting in a markedly flattened diurnal rhythm (see Fig. 4). IR and LR mice, on the other hand, did not differ very much, although LR animals tended to have lower CM levels across the 24-h light-dark cycle (see Fig. 4). These findings further support the close association between HPA axis activity/reactivity and disturbances of neuroendocrine rhythms, as for example very similar alterations, including a reduced amplitude in circadian cortisol secretion patterns, elevated trough cortisol levels and increased 24-h means, have been found in patients suffering from melancholic or psychotic depression, both of which are characterized by a strong increase in HPA axis drive [24]–[27]. Interestingly, data available for atypical depression suggest no change or a slight decrease in trough cortisol levels [7], indicating similarities with the phenotype observed in the LR mouse line (see also [30]). Although our findings match reasonably with these clinical observations, it should be highlighted that in rodents, the entire human syndrome of MD cannot be modelled, but they may share core symptoms of the disease, including the molecular pathways underlying key endophenotypes.

Potential mechanisms that might be involved in bringing about the described alterations in the circadian GC rhythm of our mouse lines include variations in the activity of neural networks (assessable as brain glucose metabolism differences across times of day) as well as abnormal levels or patterns of noradrenalin and melatonin secretion [6]–[8], [12]. Furthermore, neurodegenerative processes, particularly in structures participating in the regulation of the HPA axis such as the hippocampus, might be an important factor, as similar disturbances in the diurnal variation of GC have been reported in Alzheimer's and Parkinson's disease patients as well as in experimental models of prion disease [45]–[48]. The deterioration of the circadian rhythm is interestingly often observed before other clinical symptoms are manifested and can be indicative of a relapse in the case of MD. Therefore alterations of the circadian rhythm appear to be closely linked to the body's stress system and might have a significant impact for a number of pathologies, including MD (for reviews see [16], [49], [50]).

Genotyping efforts as well as studies addressing changes in brain neurotransmitter and neuromodulator systems (including CRH, serotonin and noradrenalin) are currently underway, shedding light on the molecular underpinnings of the endophenotypes observed in the HR/IR/LR mouse lines. Potentially, this pre-clinical research will also yield novel insights into the fundamental mechanisms involved in the pathophysiology of human diseases.

As outlined above, sleep abnormalities are very common symptoms of MD patients and have been in the focus of researchers for several decades (for reviews see [6], [7], [12], [51]–[54]). Sleep is typically divided into NREM sleep and REM sleep episodes; the former can be further subdivided into sleep stages I–IV in humans. Stage I sleep, the transition from wakefulness with its mixed frequency activity and dominant alpha waves (8–12 Hz) to shallow sleep, is marked with dominant EEG frequencies of 4–7 Hz (theta waves). Sleep spindles with frequencies of 12–15 Hz and K-complexes are hallmarks of stage II sleep [55]. In sleep stage III, delta waves with a frequency of around 1-3(4) Hz, so-called SWA, are present and become increasingly dominant in stage IV sleep (referred to as slow wave sleep). REM sleep on the other hand is characterised by a desynchronised EEG (similar to wakefulness) and episodic erratic movements of the eyes together with low amplitude electromyogram activity [55].

In healthy adults, NREM sleep and REM sleep normally alternate periodically through the night starting with around 90 min of NREM sleep, followed by a short REM sleep period of approximately 10 min. This cycle is then repeated four to six times during the night, with decreasing portions of sleep stages III and IV and increasing durations of the successive REM sleep periods towards the end of the night [56]. In depressed patients, however, increased stage I sleep, decreased stage III and stage IV sleep, shorter NREM sleep duration, insomnia (involving difficulties falling asleep, sleep fragmentation and early morning awakenings) are often reported [52], [53], [57], [58]. In addition, common sleep-EEG alterations include decreased REM sleep latency, increased REM density [59], [60] and increased total time spent in REM sleep [61]. It has to be noted, however, that these sleep alterations are not uniformly found across all MD patients. In particular, when the different subtypes of melancholic and atypical depression are considered, the emerging picture is different. Melancholic depression, for instance, is characterised by the aforementioned alterations, including poor sleep quality and decreased amounts of sleep, whereas in atypical depression poor sleep quality is rather associated with an overall increased amount of sleep and fatigue-like behaviour during the day [7], [15], [37].

Interestingly, our findings from the sleep-EEG recordings in HR, IR and LR mice also support this dichotomy of symptom clusters linked with diametral differences in HPA axis reactivity. HR mice were found to have more bouts of wakefulness during the normal resting period of the animals (see Fig. 2 and Fig. 5A) and also showed a significant reduction in the amount of NREM sleep at several experimental time points (see Fig. 5B). An extensive event related analysis (applying the ‘event-history-analysis program’ developed by Alexander Yassouridis [62]) additionally supports the notion of a shallower and more fragmented sleep in HR mice, as the number of awakenings and stage shifts, particularly from REM sleep to wake, was clearly increased during the light as well as during the dark phase in this mouse line (Fenzl and Touma et al., in preparation). These differences in sleep architecture might be attributed to the increased activation of the HPA axis across the day in the HR mouse line (see discussion above and Fig. 4). CRH is known to impair sleep and enhance vigilance, thereby suggesting a causal relationship between shallow sleep and the hyperactivity of the HPA system in melancholic depression [6]–[8], [15], [51]. Other preclinical studies also support this view. In rats, after intracerebroventricular administration of CRH, waking was enhanced, whereas alpha-helical CRH (a specific CRH receptor antagonist) reduced spontaneous waking [51], [63].

The most pronounced differences between HR, IR and LR mice, however, were found regarding the amount of REM sleep. At the majority of time points during the animals' normal resting period, HR mice spent much more time in REM sleep than the other two lines (see Fig. 5C). Human sleep data suggest that changes in REM sleep, mediated by the noradrenergic, serotonergic and cholinergic systems, are not only a consequence of depression, but can be seen as true endophenotype of the disease (reviewed in [64]). Interestingly, in a transgenic mouse model overexpressing CRH in the entire brain, REM sleep was also significantly enhanced [65], along with a clearly increased responsiveness of the HPA axis to stressors and alterations in emotional behaviour [66], hence largely overlapping with our observations in HR mice (see also [30]). Other animal studies as well as clinical findings further support the notion that CRH promotes REM sleep [6], [67], [68], although the effect of CRH on REM sleep seems to be site- and dose-dependent [8]. Moreover, our findings are in line with results of sleep investigations performed in different animal models of depression such as exposure to chronic unpredictable stress [69] and selection for increased ‘helplessness’ in the tail suspension test [70], [71]. These studies revealed very similar alterations in sleep/wake patterns, distribution of sleep stages and facilitation of REM sleep as we saw in the HR mouse line, again underlining the significant impact of stress responsiveness on sleep architecture.

In similarity to REM sleep, significant differences between HR, IR and LR mice were found in the proportion of slow wave sleep. Virtually across the entire light-dark cycle, HR mice showed a dramatically lowered level of SWA, while higher SWA was observed in LR animals (see Fig. 6). Sleep deprivation studies indicate that SWA reflects sleep intensity, as it was clearly increased as a function of waking [72]. In other words, SWA can serve as a distinct marker for homeostatic sleep pressure [73]. The regulation of SWA itself was proposed to be a function of the ‘Two Process Model’ [74], depending on the interaction of processes S (sleep dependent) and C (circadian). Sleep propensity, increasingly depending on extended time spent awake, is reflected by process S. In this model, the sleep intensity (process S) is at its maximum at sleep onset, declining during consecutive sleep. It is beyond the scope of this study to reveal whether the decreased amounts of NREM sleep can be attributed to attenuated levels of SWA, but this would implicate that reduced SWA is an intrinsic sleep-physiological feature of the HR mouse line, which might be brought about by a chronic activation of the CRH system [6], [75]. Interestingly, clinical studies also report a reduction in SWA in depressed patients [6], [7], [11], [12], [76], although slow wave sleep is not reduced and REM sleep parameters seem to be less consistently altered in patients with atypical depression [7], [17].

Taken together, our study provides clear evidence for a critical interaction between HPA axis dysregulation and rhythmicity disturbances, including changes in behavioural activity patterns, circadian GC secretion and sleep architecture. In our mouse model, hyper-responsiveness to stressors was associated with psychomotor activity alterations, resembling the restlessness, sleep discontinuity and early awakenings commonly observed in melancholic depression. Furthermore, HR mice also showed neuroendocrine abnormalities such as reduced amplitude of the circadian GC rhythm and elevated trough levels, potentially mimicking similar symptoms in MD patients. The sleep-EEG analyses revealed changes in NREM and REM sleep as well as SWA in HR mice, indicative of reduced sleep efficacy and REM disinhibition, which reasonably overlap with observations in melancholically depressed patients. Thus, by selectively breeding mice for extremes in stress reactivity, clinically relevant endophenotypes of MD can be modelled, presumably including the symptomatology and pathophysiology of specific subtypes of depression.

It should be emphasized, however, that animal models will only be able to mimic certain aspects of the human disease biology rather than the entire clinical syndrome and that not all features of our SR model match with findings in MD patients. Limitations to the clinical relevance of the HR/IR/LR mouse lines for instance include that HPA axis dysregulation is currently not one of the critical diagnostic criteria for MD and that it is a genetic model, i.e. the differences in stress responsiveness are already present early in life, thereby potentially influencing developmental processes that shape the respective endophenotypes. On the other hand, also in humans, the latter mechanisms (driven by both genetic and environmental factors) might represent key variables underlying individual vulnerability to psychiatric disorders [9], [16], [77], [78].

Therefore, we are convinced that elucidating similar aspects of biological alterations in animal models and human patients can be a major progress and that translational approaches using appropriate animal models can substantially further our understanding of how organisms respond to stress and the nature of inter-individual differences in the stress response. Given the importance of rhythmicity and sleep disturbances as biomarkers of MD, both animal and clinical studies on the interaction of behavioural, neuroendocrine and sleep parameters may reveal molecular pathways that ultimately lead to the discovery of new targets for antidepressant drugs tailored to match specific pathologies within MD.
